# Validation of myeloproliferative neoplasms associated risk factor RDW as predictor of thromboembolic complications in healthy individuals: analysis on 6849 participants of the SHIP-study

**DOI:** 10.1038/s41375-023-01943-8

**Published:** 2023-06-23

**Authors:** Kirsi Manz, Jeanette Bahr, Till Ittermann, Konstanze Döhner, Steffen Koschmieder, Tim H. Brümmendorf, Martin Griesshammer, Matthias Nauck, Henry Völzke, Florian H. Heidel

**Affiliations:** 1 Institut für Community Medicine – Abteilung Versorgungsepidemiologie und Community Health, Universitätsmedizin Greifswald, Greifswald, Germany; 2 Innere Medizin C, Universitätsmedizin Greifswald, Greifswald, Germany; 3 Institut für Community Medicine – Abteilung SHIP-KEF, Universitätsmedizin Greifswald, Greifswald, Germany; 4 German MPN Study Group, GSG-MPN, Germany; 5 Innere Medizin III, Universitätsklinikum Ulm, Ulm, Germany; 6 Innere Medizin IV, RWTH Aachen, Aachen, Germany; 7 Mühlenkreisklinikum Minden, Universitätsklinikum Bochum, Minden, Germany; 8 Institute of Clinical Chemistry and Laboratory Medicine, University Medicine Greifswald, Greifswald, Germany; 9 DZHK (German Centre for Cardiovascular Research), Partner Site Greifswald, University Medicine, Greifswald, Germany

**Keywords:** Risk factors, Translational research

## To the Editor:

Chronic myeloproliferative neoplasms (MPN) are characterized by hyperproliferation of myeloid cells leading to erythrocytosis, thrombocytosis, leukocytosis and splenomegaly. Thromboembolic events (TE) are among the most prevalent complications in patients with different subtypes of MPN such as polycythemia vera (PV) [1, 2], with arterial and venous thromboses being among the major causes of morbidity and mortality. Pathophysiologic mechanisms that contribute to TE complications, besides increased cell counts, include functional alterations of leucocytes, red blood cells, platelets and endothelial cells [3]. The rate of thromboembolic complications in MPN patients ranges from 1.1 to 4.4% per year, while this rate is significantly lower in the normal population (0.6 and 0.9% per year in the absence or presence of cardiovascular risk factors, respectively) [4, 5]. Therefore, prediction of occurrence of thromboembolic events for risk estimation is of great importance. While the risk of these patients to experience thromboembolic complications is clearly high, prognostic parameters beyond age and past history of thrombosis are currently lacking. This leads to challenges in clinical decision making regarding the indication of cytoreductive drugs and the prophylaxis and use of anticoagulants. Therefore, in our previous work, we used a machine learning algorithm to identify risk factors for this high-risk population of patients with PV for clinical use that can predict thromboembolic events [6]. Using the publicly available OPTUM database that consists of patient data provided by US insurance companies, we could define red cell distribution width (RDW), lymphocyte and platelet counts as independent prognostic parameters for thromboembolic events: Lymphocyte ratio (LYP) and RDW predicted the risk of occurrence of TEs of patients without a history of TEs within the next 12 months. In addition, predictive factors for patients with a history of TE complications included lymphocyte ratio and platelet count. Recently, neutrophil-lymphocyte ratio (NLR) was confirmed as predictive risk factor for venous thrombosis in an independent retrospective cohort of PV patients [7]. While these analyses require prospective validation in clinical trials, the predictive value of these parameters in a normal control population without myeloproliferation or hematopoietic cancers remains to be investigated.

In order to validate these findings in a control cohort of non-MPN patients, we retrieved data of the SHIP study conducted at Greifswald University Medicine. The Study of Health in Pomerania (SHIP) is a population-based epidemiological study consisting of currently 5 independent cohorts [8]. The SHIP investigates common risk factors, subclinical disorders and manifest diseases with highly innovative non-invasive methods in the population of northeast Germany. As this study is not focused on one specific disease it aims to investigate health in all aspects and complexity involving the collection and assessment of data relevant to the prevalence and incidence of common, population-relevant diseases and their risk factors.

We utilized data from different independent cohorts of the SHIP study: the baseline examination of SHIP-START (SHIP-START-0) between 1997 and 2001 (*n* = 4308), and the baseline examination of SHIP-TREND (SHIP-TREND-0) between 2008 and 2011 (*n* = 4420). After excluding missing datapoints, a total of 2491 datasets (derived from individual participants) from the SHIP-START-0 and 4358 from the SHIP-TREND-0 were included in the analyses. Data on all probands with baseline data on RDW, lymphocyte percentage, platelet count, body mass index (BMI), prior TE, neutrophil percentage, leukocytes and hematocrit was used for the study. Also, all documented medication was recorded and included for analysis. Of note, SHIP-START-0 data does not include differential blood counts, including lymphocyte and neutrophil percentage. Occurrence of TE in SHIP-START was defined as thrombosis, stroke or myocardial infarction or use of an antithrombotic agent while SHIP-TREND also included evidence of thrombophlebitis. Antithrombotic agents were defined as agents belonging to the Anatomical Therapeutic Chemical (ATC) classification system section B01 “antithrombotic agents”. This section includes oral anticoagulants such as vitamin K antagonists, platelet inhibitors (ASA and P2Y-antagonists) and direct oral anticoagulants (DOACs) among others. Single use of antithrombotic agents e.g. for in-flight prophylaxis was not considered. Cardiovascular risk factors included were elevated blood lipids, hypertension, diabetes mellitus, current smoking, BMI, and subjects’ age. Subjects with missing diabetes mellitus status and HbA1c > = 6.5% were counted as diabetic. In the absence of elevated blood lipid status, subjects with cholesterol > = 6 mmol/l and/or triglyceride > 1.9 mmol/l were assigned to elevated blood lipids. Descriptive statistics are provided as median and minimum - maximum, or as frequency and percentage, as appropriate. The non-parametric Mann Whitney U test was used to assess differences of continuous variables between two groups. Categorical variables were compared using the Fisher’s exact test. First, all candidate variables were adjusted for age and sex. Then, all significant age- and sex-adjusted variables were included in the backward variable selection procedure. Variable selection was performed 1000 times using bootstrapping methods. To report the most relevant variables, the final model consists of those selected in at least 80% of the bootstrapping runs. In both cohorts, 70% of the data were used to build and 30% were used to validate the model. To assess the predictive value of both models, accuracy and receiver operating characteristic (ROC) curve were calculated. Statistical significance was claimed at 5% (*p* < 0.05) and no correction for multiple testing was performed. The data was prepared using SAS 9.4 (SAS Institute Inc., Cary, NC, USA) and analyzed using R Version 4.2.2 [9].

Regarding baseline characteristics, TE events had occurred in 321 (12.9%) of the 2491 individuals of the SHIP-START cohort while the prevalence of TE events was 21.4% (932 events in 4358 individuals) in the SHIP-TREND cohort. Overall, established risk factors for TE such as male sex, higher age, higher body-mass-index (BMI), arterial hypertension, hypercholesterolemia, and diabetes mellitus were associated with significantly higher rate of TE events (Table 1). Of note, TE events were more frequently reported in non-smokers compared to smokers in both cohorts. In regard to laboratory parameters, higher RDW and lower platelet counts showed significant association with TE complications. In contrast, higher leukocyte counts, lower hematocrit and lower lymphocyte ratio showed exclusively significance in the SHIP-TREND cohort analysis.

**Table 1:** Baseline characteristics of both cohorts.

	SHIP-START-0				SHIP-TREND-0			
	Overall (*N* = 2491)	No TE event (*N* = 2170)	TE event (*N* = 321)	*p* value	Overall (*N* = 4358)	No TE event (*N* = 3426)	TE event (*N* = 932)	*p* value
**Sex**								
Female	1258 (50.5%)	1146 (52.8%)	112 (34.9%)	**<0.0001**	2245 (51.5%)	1808 (52.8%)	437 (46.9%)	**0.0015**
Male	1233 (49.5%)	1024 (47.2%)	209 (65.1%)		2113 (48.5%)	1618 (47.2%)	495 (53.1%)	
**Age** (years)	49 [20,81]	46 [20,81]	67 [20,80]	**<0.0001**	52 [20,84]	48 [20,82]	66 [22,84]	**<0.0001**
**BMI** (kg/m^2^)	27.1 [16.1,58.4]	26.7 [16.1,47.7]	28.9 [18.4,58.4]	**<0.0001**	27.5 [15.4,54.4]	27.0 [15.4,54.4]	29.3 [17.7,51.2]	**<0.0001**
**Hypercholesterolemia**								
No	1834 (73.6%)	1664 (76.7%)	170 (53.0%)	**<0.0001**	3432 (78.8%)	2883 (84.2%)	549 (58.9%)	**<0.0001**
Yes	657 (26.4%)	506 (23.3%)	151 (47.0%)		926 (21.2%)	543 (15.8%)	383 (41.1%)	
**Hypertension**								
No	1473 (59.1%)	1353 (62.4%)	120 (37.4%)	**<0.0001**	2264 (52.0%)	1980 (57.8%)	284 (30.5%)	**<0.0001**
Yes	1018 (40.9%)	817 (37.6%)	201 (62.6%)		2094 (48.0%)	1446 (42.2%)	648 (69.5%)	
**Diabetes mellitus**								
No	2291 (92.0%)	2039 (94.0%)	252 (78.5%)	**<0.0001**	3904 (89.6%)	3179 (92.8%)	725 (77.8%)	**<0.0001**
Yes	200 (8.0%)	131 (6.0%)	69 (21.5%)		454 (10.4%)	247 (7.2%)	207 (22.2%)	
**Smoking**								
No	1762 (70.7%)	1482 (68.3%)	280 (87.2%)	**<0.0001**	3184 (73.1%)	2415 (70.5%)	769 (82.5%)	**<0.0001**
Yes	729 (29.3%)	688 (31.7%)	41 (12.8%)		1174 (26.9%)	1011 (29.5%)	163 (17.5%)	
**RDW** (%)	12.4 [10.8,27.7]	12.3 [10.8,23.9]	12.8 [10.9,27.7]	**<0.0001**	13.1 [11.3,21.9]	13.1 [11.3,21.9]	13.4 [11.8,20.5]	**<0.0001**
**Platelets** (10^9^/l)	226 [40, 828]	224 [59, 566]	207 [40, 828]	**<0.0001**	223 [43, 576]	227 [43, 576]	213 [51,502]	**<0.0001**
**Leukocytes** (10^9^/l)	6.4 [2.0,34.6]	6.4 [2.0,34.6]	6.3 [2.8,14.1]	0.8314	5.8 [2.3,117.0]	5.8 [2.3,117.0]	6.0 [2.6,15.6]	**0.0001**
**Hematocrit** (%)	39.9 [26.0,56.0]	39.9 [26.0,56.0]	40.0 [29.4,51.0]	0.3683	41.8 [28.3,58.7]	41.9 [28.3,58.7]	41.5 [28.8,54.5]	**0.0070**
**LYP** (%)	NA				29.2 [4.7,95.2]	29.6 [4.7,95.2]	27.6 [6.8,49.8]	**<0.0001**

Shown is median [minimum, maximum] for continuous and frequency (%) for categorical variables. *p* value is for the comparison of patients with TE vs. without TE.

*TE* thromboembolic event, *BMI* body mass index, *RDW* red cell distribution width, *LYP* lymphocyte ratio (lymphocyte percentage).

Statistically significant values are highlighted in bold.

To assess for effects of the above risk factors on TE events, we used multivariable logistic regression models. When investigating the SHIP-START cohort of 2491 individuals, male sex (*p* < 0.0001), presence of hypertension (*p* = 0.0042), hypercholesterolemia (*p* < 0.0001) or diabetes mellitus (*p* = 0.0008), and higher age (*p* < 0.0001) were validated as TE risk factors. Regarding laboratory parameters, higher RDW (*p* = 0.0006) was the only predictor for TE complications.

Analysis of the SHIP-TREND cohort of 4358 individuals confirmed independent predictive value of higher age (*p* < 0.0001) and hypercholesterolemia (*p* < 0.0001) while elevated body mass index (BMI; *p* = 0.0003) scored as an additional predictive factor due to availability of the respective data points in this cohort. In contrast, male sex and hypertension were not confirmed as independent risk factors. Consistent with the SHIP-START cohort, higher RDW (*p* < 0.0001) was identified as predictive for TE events, along with lower platelet counts (*p* < 0.0028). Taken together, alterations of laboratory parameters such as red cell distribution width and platelet count at study entry were associated with occurrence of thromboembolic events in this retrospective assessment of individuals without evidence for hematologic malignancies.

**Fig. 1:**
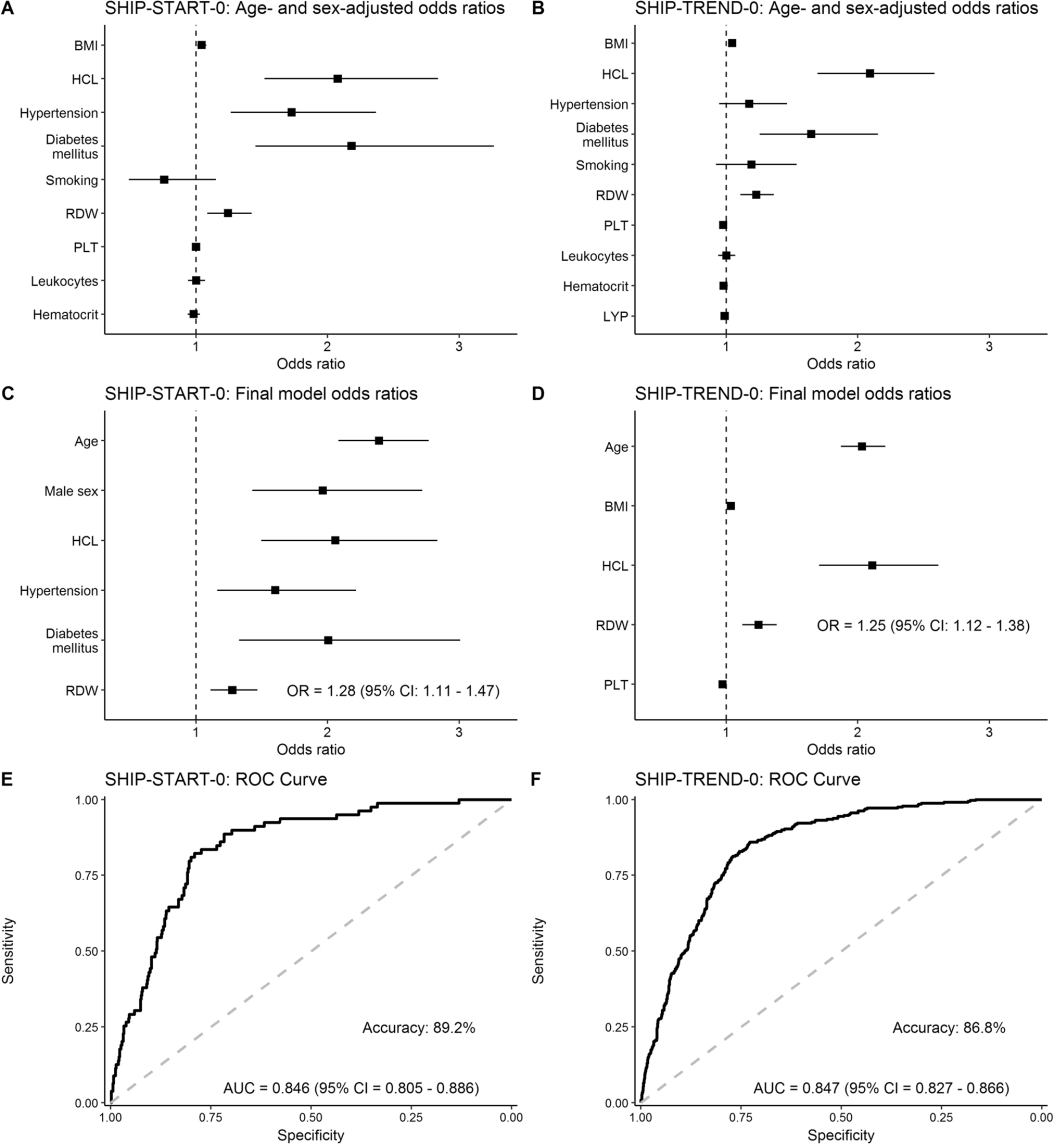
Effects of risk factors on TE events. Age- and sex-adjusted odds ratios (**A**, **B**), final model odds ratios (**C**, **D**) and receiver operating characteristic (ROC) curve (**E**, **F**) for SHIP-START-0 data (**E**) and SHIP-TREND-0 data (**F**). BMI body mass index, HCL hypercholesterolemia, RDW red cell distribution width, PLT platelet count, LYP lymphocyte ratio, AUC area under curve, CI confidence interval.

When adjusting for age and sex (Fig. 1A, B), BMI, hypercholesterolemia, hypertension, diabetes mellitus and RDW consistently showed elevated odds ratios in both cohorts, using the basic model. Assessment for TE risk factors in the final model confirmed age, hypercholesterolemia and RDW as predictors of thromboembolic events in both cohorts. Here, RDW showed an OR of 1.28 (95% CI: 1.11–1.47) for SHIP-START and 1.25 (95% CI 1.12–1.38) for SHIP TREND (Fig. 1C, D). Of note, the SHIP-TREND model could also be validated using SHIP-START data. In order to select an optimal model, receiver operating characteristic (ROC) analysis was performed showing an AUC of 0.846 (95% CI: 0.805–0.886) for SHIP-START and an AUC of 0.847 (95% CI: 0.827–0.866) for SHIP-TREND (Fig. 1E, F). Accuracy of the SHIP-START model was 89.2% and of the SHIP-Trend model 86.8%.

Red cell distribution width is a marker for the variation of erythrocyte size (anisocytosis) and used in combination with other laboratory markers for differential diagnosis of hematological diseases such as anemia and bone marrow dysfunction. Changes in RDW have been reported for a variety of chronic inflammatory conditions such as diabetes, cardiovascular disease, infections and cancer and its predictive and prognostic value has been reported for cardiovascular disease as well as for overall mortality of the general population [10]. Likewise, differential blood counts have been described as biomarkers of inflammatory processes and cancers. Identification of RDW, platelet counts and lymphocyte ratio as biomarkers for thromboembolic events in PV patients is therefore not surprising, as JAK2-mutated cancers are associated with broad activation of cell signaling [11] and increase of pro-inflammatory cytokines [3, 12]. Recently, exome-analysis studies have shown age-related clonal hematopoiesis (CH) in healthy individuals, driven by mutations of genes recurrently mutated in myeloid neoplasms and associated with an increased risk of hematologic cancer and cardiovascular disease. Critically, both SHIP-cohorts reported in this analysis have not been investigated for the presence of clonal hematopoiesis. Therefore, we cannot exclude the influence of CH on the predictive value of RDW and occurrence of TE events. Moreover, cutoffs for RDW may vary and have not been generally defined in previous analyses. Critical limit values of these potential biomarkers may depend on the underlying condition, comorbidities (e.g. previous TE complications) or concomitant medication. Finally, in SHIP-TREND, we used a broad definition of TE events (including peripheral thrombosis and thrombophlebitis) and predictive biomarker values may vary with a definition restricted to deep vein thrombosis, pulmonary embolism, myocardial infarction and stroke. Of note, the allocation of individuals into the “TE-event” cohort based on the use of anticoagulants may result in inclusion of individual participants using ASA and P2Y-antagonists as primary rather than secondary prophylaxis.

Taken together, we could confirm RDW as an independent predictive parameter for thromboembolic events in the general population. Development and prospective validation of predictive scoring systems combining predictive laboratory parameters are clearly warranted but are beyond the scope of this report.
